# PoplarGene: poplar gene network and resource for mining functional information for genes from woody plants

**DOI:** 10.1038/srep31356

**Published:** 2016-08-12

**Authors:** Qi Liu, Changjun Ding, Yanguang Chu, Jiafei Chen, Weixi Zhang, Bingyu Zhang, Qinjun Huang, Xiaohua Su

**Affiliations:** 1 State Key Laboratory of Tree Genetics and Breeding, Research Institute of Forestry, Chinese Academy of Forestry, Key Laboratory of Tree Breeding and Cultivation, State Forestry Administration, Beijing 100091, China; 2 Co-Innovation Center for Sustainable Forestry in Southern China, Nanjing Forestry University, Nanjing 210037, China.

## Abstract

Poplar is not only an important resource for the production of paper, timber and other wood-based products, but it has also emerged as an ideal model system for studying woody plants. To better understand the biological processes underlying various traits in poplar, e.g., wood development, a comprehensive functional gene interaction network is highly needed. Here, we constructed a genome-wide functional gene network for poplar (covering ~70% of the 41,335 poplar genes) and created the network web service PoplarGene, offering comprehensive functional interactions and extensive poplar gene functional annotations. PoplarGene incorporates two network-based gene prioritization algorithms, neighborhood-based prioritization and context-based prioritization, which can be used to perform gene prioritization in a complementary manner. Furthermore, the co-functional information in PoplarGene can be applied to other woody plant proteomes with high efficiency via orthology transfer. In addition to poplar gene sequences, the webserver also accepts Arabidopsis reference gene as input to guide the search for novel candidate functional genes in PoplarGene. We believe that PoplarGene (http://bioinformatics.caf.ac.cn/PoplarGene and http://124.127.201.25/PoplarGene) will greatly benefit the research community, facilitating studies of poplar and other woody plants.

Woody plants, especially long-lived forest trees, provide large amounts of biomass, serving as vital raw materials for renewable energy production and other valuable commercial products. However, due to the long lifecycles of these plants, many of which have relatively large genomes, it is difficult to perform experiments using these plants, which has motivated the development of a model woody plant system^1^. Poplar has several attributes that have led to its emergence as such a model system, including rapid growth, ease of clonal propagation, relatively small genome, easy transformation and so on^2^,^3^. Understanding the characteristics of poplar, including various developmental processes, such as growth and wood development, will great facilitate the study of long-lived, large perennial plants. Although poplar is the first woody plant whose complete genome has been sequenced, and dozens of genes encoding poplar traits have been identified, functional knowledge about these genes and the genetic factors underlying these traits remains limit. Recent advances in high-throughput sequencing^4^, such as RNA-seq-based transcriptome studies and re-sequencing-based genetics studies, have generated unprecedented amounts of functional genomics data associated with many traits in poplar^5^,^6^, which greatly facilitates the study of many important traits of poplar genome-wide.

The regulation of biological processes involves networks of various genes that function in a complex, coordinated manner. However, to date, most studies of poplar have focused on only a single or limited number of genes. Although gene coexpression networks have been constructed to identify functional gene modules involved in the conditions of interest^7^,^8^,^9^,^10^,^11^, no comprehensive functional network of the interactome of poplar is currently available, and there is a strong demand for such public web resources. Functional gene interaction networks serve as powerful tools for gene functional linkage studies in many organisms including animals, plants and prokaryotes^12^,^13^,^14^. Among the functional network construction algorithms, the development of probabilistic functional gene networks increases both network accuracy and coverage by integrating heterogeneous biological data into a single model^15^,^16^. Using this approach, functional associations are determined between genes in a genome based on diverse data sets, each containing millions of individual observations, which are then integrated into a comprehensive gene network. Once the comprehensive functional linkage network is generated, genes whose functions are unknown could easily be annotated based on their linkage to genes with known functions. In addition, network-guided screening could be performed to identify new candidate genes linked to a specific trait based upon network linkages with previously identified genes associated with these traits.

Here, we constructed a genome-wide co-functional gene network for poplar (covering ~70% of the 41,335 *Populus trichocarpa* coding genome) based on machine learning technologies and created a network web service, PoplarGene, offering numerous functional interactions and extensive poplar gene functional annotations. PoplarGene incorporates two network-assisted gene prioritization algorithms, neighborhood-based prioritization^17^ and context-based prioritization^18^, which can be used to perform gene prioritization and to identify genes underlying traits in a complementary manner. Additionally, the co-functional linkage information in PoplarGene can be utilized for other woody plant proteomes via orthology transfer using two optional orthology mapping algorithms (Bidirectional Best Hits^19^,^20^ and InParanoid^21^). In addition to poplar genes, the webserver also accepts Arabidopsis reference genes as input to guide the search for novel candidate functional genes in the PoplarGene network. We found that PoplarGene has significant predictive power for identifying genes affecting specific traits, such as secondary xylem development, stress response and defense genes. To the best of our knowledge, PoplarGene is the most comprehensive functional linkage resource for poplar to date. We believe that its user-friendly web interface will be highly beneficial to the research community, representing a valuable resource for better understanding poplar and other woody plants.

## Results and Discussion

### Network construction

**Figure 1:**
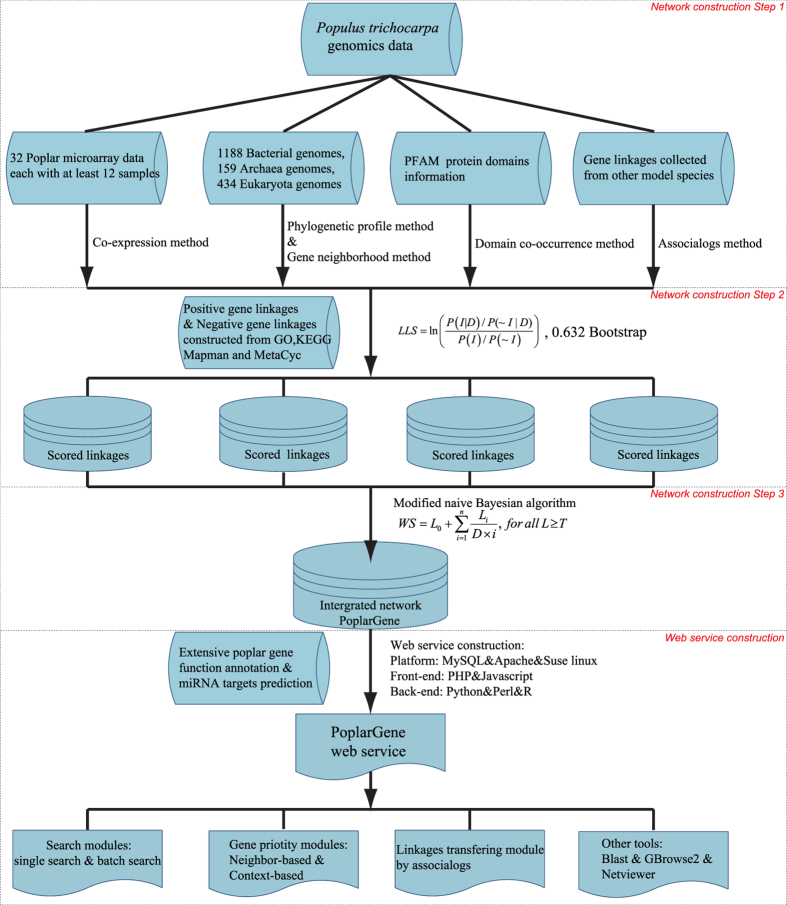
The overall workflow of PoplarGene construction. PoplarGene network construction included three main steps: (**a**) inferring functional gene pairs; (**b**) assigning likelihood ratio scores for network links and (**c**) integrating component network linkages. The PoplarGene web server was then developed based on network linkages and other related functionalities.

**Figure 2:**
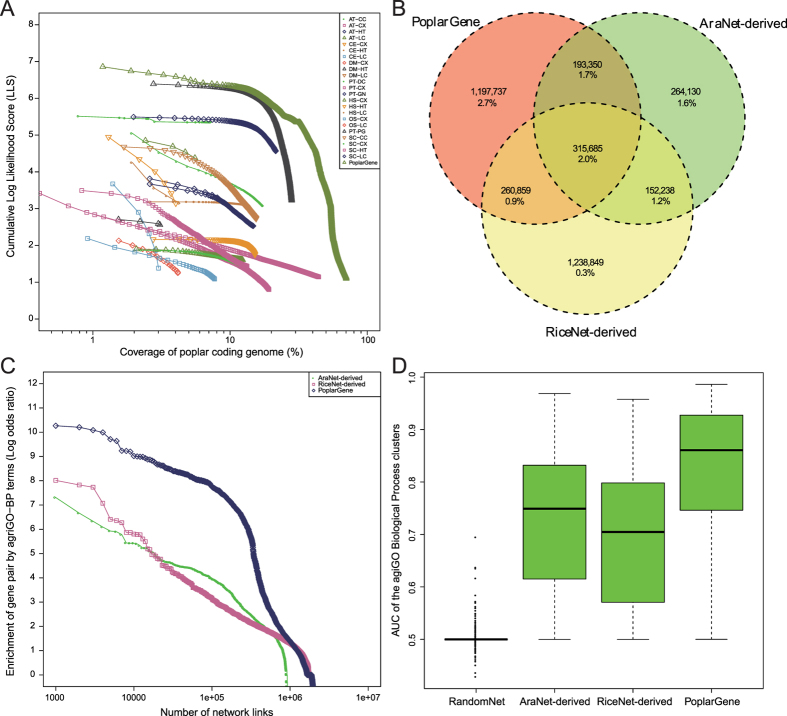
Summary of quality assessment of the PoplarGene network. (**A**) The gene linkages derived from 23 diverse functional genomics data sets, representing millions of experimental or computational observations, were integrated into a comprehensive network with higher accuracy and genome coverage than any single data set. The integrated network contains 1,967,631 linkages and 29,049 genes (>70% of the *P*. *trichocarpa* coding genome). The x-axis represents the log-scaled coverage of the *P*. *trichocarpa* coding genome covered by linkages derived from the corresponding datasets (curves). The y-axis indicates the accuracy of functional linkages, measured as the cumulative log likelihood of linked genes to shared GO-BP term annotations tested using 0.632 bootstrapping and plotted for each bin of 1,000 linkages. The datasets were designated AA-BB, with AA indicating species of data origin (AT, *A*. *thaliana*; CE, *C*. *elegans*; DM, *D*. *melanogaster*; HS, *H*. *sapiens*; OS, *O*. *sativa*; PT, *P*. *trichocarpa*; SC, *S*. *cerevisiae*) and BB indicating data type (CC, co-citation; CX, mRNA coexpression; DC, domain co-occurrence; GN, gene neighbor; LC, literature curated protein interactions; HT, high-throughput experimental screening of interaction; PG, phylogenetic profiles). (**B)** Venn diagram of the gene linkages, indicating that the PoplarGene network contains many more linkages than those derived by orthology transfer from the Arabidopsis gene network AraNet^12^ and the rice gene network RiceNet^32^ and that they have higher linkage accuracy. Linkage accuracy was measured using an independent set of reference linkages obtained from the agriGO database. (**C**) Precision-recall analysis comparing the PoplarGene network to the AraNet-derived network and the RiceNet-derived network. (**D**) Box-and-whisker plot of network predictive power for 277 agriGO BP terms (with more than four annotated genes), as measured by the area under the curve from ROC analysis.

The PoplarGene network was constructed based on diverse types of large-scale experimental and genomic datasets using machine-learning methods (Fig. 1). Three major steps were involved in PoplarGene network construction: (a) inferring functional gene pairs from each experimental and genomic dataset; (b) assigning likelihood ratio scores for each network linkage benchmark using gold-standard gene pairs and (c) integrating component network linkages using a modified naive Bayesian algorithm. Network construction was based on the *Populus trichocarpa* v3.0 reference genome obtained from Phytozome v10.3^22^, which contains 41,335 protein-coding genes. The gold-standard functional gene pairs used for network training were derived from Biological Process of Gene Ontology in Biofuel Feedstock Genomics Resource (BFGR)^23^, KEGG pathway^24^, MapMan Pathway^25^ and PoplarCyc pathway^26^. We obtained a total of 961,462 positive and 72,756,688 negative gold-standard gene linkage pairs, which were then used as the training set in a Bayesian framework^27^ to measure the likelihood of functional links between two genes. We performed the training for each type of dataset, generating a total of 23 component networks (Table 1), which were integrated into a single comprehensive network using the weighted sum strategy^28^. The integrated network contains 29,049 genes (covering >70% of the *P*. *trichocarpa* proteome) and 1,967,631 linkages. Precision-Recall analysis^29^, in which, gene pairs were ranked by LLS score, and cumulative precision and recall were then calculated with successive bins of 1,000 gene pairs, indicated that the integration improved both genome coverage and linkage accuracy compared to all datasets alone (Fig. 2A).

### Network validation

To validate the accuracy of the constructed network, GO-BP terms from the agriGO database were utilized^30^. This GO annotation set is alternative from BFGR GO-BP, which was used in our previous gold-standard training data construction. To avoid validation bias towards the broad GO-BP terms, the top 12 broadest terms in GO-BP were excluded from agriGO. We ultimately obtained 247,285 positive and 18,238,543 negative validated gene linkage pairs, overlapping 8% of our gold-standard training-positive gene pairs. Meanwhile, we also used the gene pair set derived from agriGO “Cellular Component” ontology terms as an additional benchmark set (220,946 positive and 2,465,233 negative), approximately 4% and 2% of which overlap with BFGR GO-BP-based gene pairs and gold-standard training-positive gene pairs, respectively. One important way to construct a poplar gene network is to perform orthology transfer of linkages from the existing Arabidopsis and rice comprehensive functional gene networks using associalogs methods^31^. First, to assess the accuracy of our network, we generated an AraNet-derived network and RiceNet-derived network by transferring the linkages from AraNet^12^ and RiceNet^32^, respectively. The comparison between the PoplarGene network, AraNet-derived poplar network and RiceNet-derived poplar network demonstrated that the PoplarGene network not only has larger genome coverage (number of genes in the network), but it also has higher linkage accuracy, as assessed using the validated gene pairs (Fig. 2B). Precision-Recall (PR) analysis^29^ further revealed that logarithmic OR ratios across high-scoring network linkages were higher than those of the AraNet-derived network and RiceNet-derived network (Fig. 2C). PR analysis using GO-CC-based benchmark sets also supported the same conclusion (Supplementary Figure S2A), confirming the improved accuracy and coverage of the PoplarGene network.

Second, we used several types of network property computational analyses to evaluate the quality of the PoplarGene network for biological process modeling. Power-law degree distribution analysis^33^ indicated that, like other large-scale biological system networks, the PoplarGene network is also a scale-free network (Supplementary Figure S1A)^34^. We then conducted topological analysis to assess the consistency between network modular structures and well-defined biological processes. The result show that the clustering coefficient of PoplarGene was ~200-fold higher than that of a random network (Supplementary Figure S1B), which is an expected property of functional modules comprising a network^33^. Moreover, the non-randomness of the shortest path lengths between gene pairs in PoplarGene indicates that tightly interconnected functional modules are separated by long functional links (Supplementary Figure S1C). Together, the network properties analyses revealed the gene module organization in the PoplarGene network.

Third, we used guilt-by-association (GBA) analysis^17^ to determine whether known biological pathways could be detected by the network modules in PoplarGene^35^. Candidate genes in the network were prioritized based on the direct network links to known genes (guide genes) in each biological process^17^,^36^. We evaluated the predictive power for candidate gene function for each biological process by leave-one-out cross-validation and receiver operating characteristic (ROC) analysis^37^. Tightly interconnected biological process member genes would be highly ranked based on high network prediction power, as indicated by high AUC (area under the ROC curve, 0.5 for random expectation and 1 for perfect prediction)^38^. We tested the predictive power of 277 agriGO Biological Process terms with more than four annotated genes^30^. The results reveal that PoplarGene has much higher predictive power for diverse biological pathways than random-chance expectation (P = 2.2 × e^−16^, Wilcoxon signed rank test; Fig. 2D). Moreover, PoplarGene had significantly higher AUC scores than both the AraNet-derived network (P = 3.606 × e^−14^, Wilcoxon signed rank test) and the RiceNet-derived network (P = 2.2 × e^−16^, Wilcoxon signed rank test), indicating that the PoplarGene network is highly predictive of gene function (Fig. 2D). The analysis using agriGO-CC-derived benchmark sets also supported this conclusion (Supplementary Figure S2B).

### PoplarGene web service

#### Implementation

**Figure 3:**
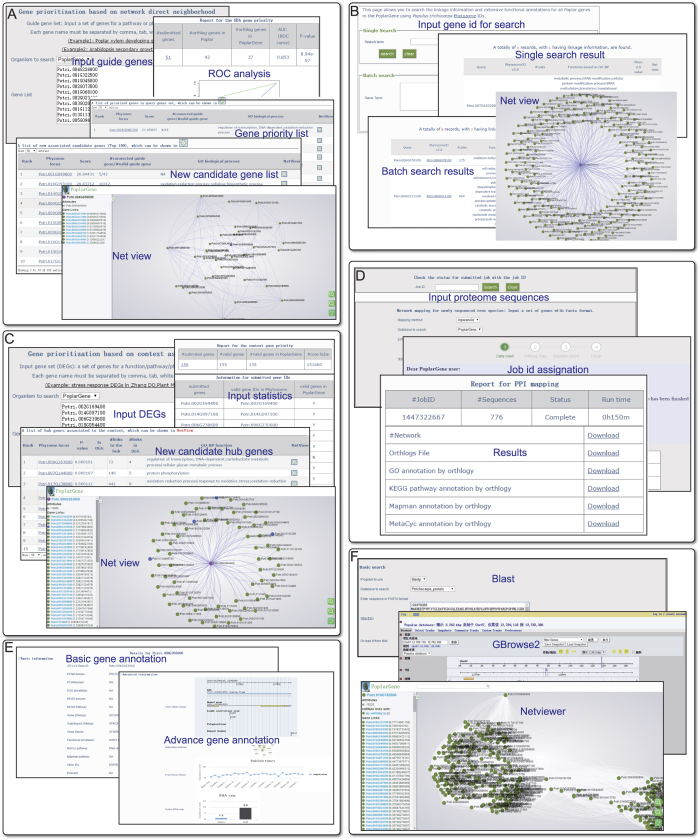
Screenshots of the PoplarGene web service. Five modules were included in the PoplarGene web service: (**A**) Neighborhood-based gene priority module; (**B**) Gene search module; (**C**) Context-based gene priority module; (**D**) Interaction transferring module and (**E**) Gene extensive annotation module. (**F**) Other tools provided by the PoplarGene web service.

The PoplarGene web service (http://bioinformatics.caf.ac.cn/PoplarGene and http://124.127.201.25/PoplarGene) is hosted on the Apache/PHP/MySQL environment under a Linux system and is equipped with two Octa-cores AMD processors (2.6 GHz each) and 64 GB of RAM. The back-end pipeline is implemented in the Python/Perl language, and the plots are drawn by R (http://www.r-project.org) and JavaScript. Network nodes and edges were stored and organized in Neo4j (http://neo4j.com/), a highly scalable native graph database management system that was specifically designed to host graphical data. An integrated network exploration JavaScript library, sigma.js (http://sigmajs.org/), was used for network graph drawing. The web interfaces were successfully tested on different web browsers, including Mozilla Firefox 42.0, Google Chrome 47.0, Safari 5.1.10 and Internet Explorer 11.0. The PoplarGene web service provides users with very user-friendly interfaces for performing gene querying and other extensive network analysis functions (Fig. 3).

### Network-assisted gene prioritization

An effective strategy for genetic dissection of complex traits is network-assisted gene prioritization^17^,^18^,^32^. To better utilize network linkage information and publicly available poplar gene-to-phenotype association information, PoplarGene offers two complementary methods to conduct network-assisted gene prioritizations for specific phenotypes. In addition, the web service can accept guide gene input from Arabidopsis, allowing the user to benefit from the available functional information about the most extensively studied plant species.

The first network-assisted gene prioritization method is neighborhood-based gene prioritization^17^, which is based on direct neighborhoods in the network (Fig. 3A). This method prioritizes new candidate genes for a specific phenotype by weighting (sum of edge LLS [Log likelihood score] weights) the direct connection to know genes involved in the phenotype (guide genes, submitted by the user). The server lists the top 100 novel candidate genes for the specific phenotype; the full list of ranked candidate genes is also available on the Results webpage. In addition, the AUC score, representing the predictive power for the submitted guide genes, is calculated using ROC analysis and is reported on the Results webpage as well. AUC ranges from 0.5 for random chance expectation to 1.0 for perfect predictions; AUC > 0.7 indicates good predictive power.

The second network-assisted gene prioritization method in the PoplarGene web service is based on a context-centric approach (Fig. 3C)^18^. Due to the long reproductive cycle and less efficient transformation procedures in poplar functional studies, the number of known guide genes for numerous poplar traits is still very limited, which hinders the efficient utilization of neighborhood-based gene prioritization. Transcriptomic analysis, largely facilitated by high-throughput sequencing in recent years, has become an efficient alternative approach to studying gene-to-phenotype associations. However, many differentially expressed genes (DEGs) identified in transcriptome studies are not actual regulatory genes but are simply genes that respond to alterations in cellular state. Moreover, many genes associated with a particular phenotype are not significantly differentially expressed. PoplarGene can prioritize genes using DEGs from a specific biological context. We initially identified 15,004 central hub genes with no less than 50 directly connected neighbors in the PoplarGene network. Users can initiate the analysis by submitting a set of DEGs that are associated with a specific biological context. Central hub genes that are significantly associated with the biological context will be returned and are subjected to Fisher’s exact test to evaluate the statistical enrichment of the neighbors of central hubs among the DEGs.

### Mapping functional links to other tree species based on orthology

The PoplarGene web service also provides a feasible and convenient way to construct genome-scale gene functional networks for other woody plants based on proteome sequence data (Fig. 3D). Three gene functional network templates (AraNet v2, RiceNet v2 and PoplarGene) and two orthology mapping algorithms (Bidirectional Best Hit^19^,^20^ and InParanoid^21^) are supported in PoplarGene. The web service also performs functional annotations for the submitted proteome using four pathway annotation systems (GO-BP, KEGG pathway, MapMan pathway and MetaCyc pathway) simultaneously. Once users successfully submit the proteome sequences, the web service will give the users a job ID, which can be used to retrieve the results once the job is completed.

### Other functionalities in PoplarGene

All poplar genes (*P*. *trichocarpa* v3.0 reference genome) are extensively annotated in the PoplarGene web service, including their pathway annotation, protein domain annotation, orthology annotation, expression atlas, expression profile in woody plant tissues (Fig. 3E) and so on. All poplar gene information can be retrieved via user-friendly search interfaces, including single gene search mode and batch gene search mode (Fig. 3B). The linkages of each gene are also downloadable in SIF format which could serve as the input for Cytoscape software (http://www.cytoscape.org/download.php) installed on local desktop computers. Additionally, the functions of query genes whose functions are unknown can be inferred from network neighbors based on GO-BP term annotations. The functional terms for the query genes are assigned based on directly connected network neighbors with GO-BP annotations and are ranked using the sum of the edge LLS weight scores. Top ten GO-BP terms will be returned as candidate functions for the query gene. In addition, poplar microRNA target binding information, BLAST search functions, GBrowse2 (http://gmod.org/wiki/GBrowse), Jbrowse (http://jbrowse.org/) and Netviewer (based on Sigma.js) tools are also available at the PoplarGene web service (Fig. 3F).

### Case studies

**Figure 4:**
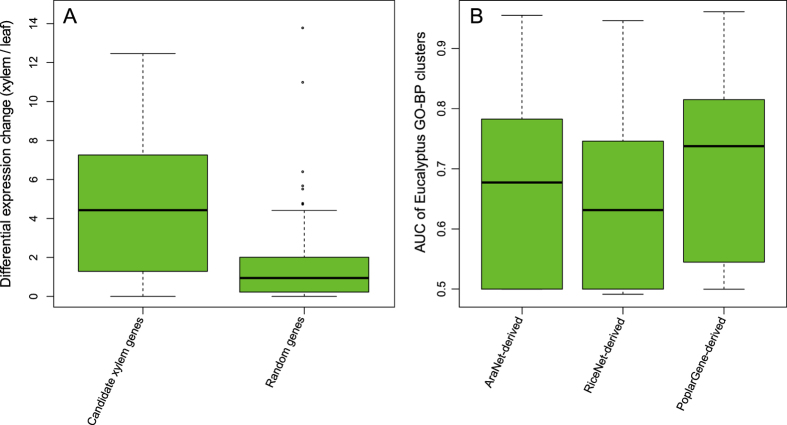
Case studies using PoplarGene. (**A**) Validation of new candidate poplar genes for secondary xylem development based on the neighborhood-based gene priority method. (**B**) Orthology transfer of PoplarGene network linkage to *Eucalyptus grandis*.

The number of poplar genes annotated using experimental evidence is quite limited, whereas Arabidopsis has the most extensive functional information of any plant. Wood is a complex structure, and thousands of genes have been shown to be associated with wood development in many species^39^,^40^,^41^,^42^. A large number of genes associated with wood/xylem development in Poplar remain unknown. Thus, an effective approach is to prioritize novel poplar genes for xylem development using Arabidopsis orthologs for the equivalent trait. The likelihood of the new candidates could be validated based on tissue-specific expression patterns, assuming that genes for xylem development exhibit more active changes in expression in xylem than in leaf tissue. We submitted 50 Arabidopsis genes known to control xylem cell specification for neighborhood-based gene prioritization in the PoplarGene web service (see Supplementary Figure S3A for the workflow), which returned 2,399 new candidate poplar genes. We then used poplar RNA-seq transcriptome data (Sequence Read Archive ID: SRP050172)^5^, which were obtained from a comparative study of gene expression in xylem and leaf tissue, to validate the new candidate genes. The top 100 candidate genes were significantly more differentially expressed in xylem versus leaf tissue than 100 randomly selected poplar genes (P = 5.2 × e^−10^, Wilcoxon rank sum test; Fig. 4A).

We then used context-based gene prioritization in PoplarGene to prioritize poplar genes for defense response and stress response traits. First, we submitted 155 stress-responsive poplar DEGs^43^ to PoplarGene and identified 474 context-associated hubs as new candidate genes (P ≤ 0.01, Fisher’s exact test) (Supplementary Figure S3B). To validate the predictions, we measured the enrichment of 1,035 genes related to stress responses annotated by Gramene^44^ GO-BP terms among the predicted 474 genes, revealing significant enrichment of the annotated stress response genes among the new candidate genes (P = 1.347 × e^−11^, Fisher’s exact test). Second, we submitted 55 poplar defense DEGs^45^,^46^ to PoplarGene, which returned a total of 367 context-associated hubs as new candidate genes (P ≤ 0.01, Fisher’s exact test). We then used 841 genes related to defense responses annotated by Gramene^44^ GO-BP terms to measure enrichment of the predicted 367 genes. The results also reveal significant enrichment of the annotated defense response genes among the new candidates (P = 0.019, Fisher’s exact test).

To evaluate orthology-transferred functional gene networks for other woody plants using PoplarGene, we constructed *Eucalyptus grandis* functional gene networks based on AraNet, RiceNet and PoplarGene (Supplementary Figure S3C), which generated 483,742 linkages (14,036 genes), 950,409 linkages (13,844 genes) and 1,328,017 linkages (17,093 genes), respectively. The qualities of the transferred networks were assessed using GO-BP term recovering analysis based on the areas under Receiver Operating Characteristic curves. A total of 310 GO-BP terms (≥5 members) from the *E*. *grandis* coding-sequence genome annotated by Phytozome v10.3 were used for this analysis. The results demonstrate that AUC scores of PoplarGene-derived *E*. *grandis* network significantly outperformed both the AraNet-derived *E*. *grandis* network (P-value = 3.61 × e^−14^, Wilcoxon rank sum test) and the RiceNet-derived *E*. *grandis* network (P-value = 2.20 × e^−16^, Wilcoxon rank sum test; Fig. 4B).

In Poplar, *PtrWND2B* (Potri.002G178700) interacts with PtrVND/SND genes to regulate several poplar R2R3 MYB genes involved in secondary cell wall biosynthesis^47^,^48^. In the PoplarGene networks, we found that *PtrWND2B* has functional links with 15 genes (Potri.013G113100, VND7; Potri.005G096600, MYB63; Potri.017G016700, SND2; Potri.004G207600, XCP1; Potri.001G099800, MYB103; Potri.009G061500, MYB83; Potri.001G112200, KNAT7; Potri.007G135300, SND2; Potri.005G063200, MYB69; Potri.019G083600, VND7; Potri.003G132000, MYB103; Potri.001G197000, MYB26; Potri.003G022800, XND1; Potri.006G122100, MYB27; Potri.004G086300, MYB43). Among these linked genes, eight genes are MYB genes and Potri.005G096600 (PtrMYB028/MYB63), Potri.009G061500 (PtrMYB020/MYB83) and Potri.004G086300 (PtrMYB018/MYB43) were reported to be directly link to *PtrWND2B* by experimental study^47^.

## Conclusion

In this study, we constructed a functional gene network of poplar from diverse data sources using machine-learning procedures, which improved both the genome coverage and linkage accuracy. We then developed the PoplarGene web service, a publicly available gene network resource and network-assisted gene prioritization service that provides the poplar community with a number of useful functions. We demonstrated that not only can PoplarGene be used to predict the functions of unknown genes and to predict new candidate genes affecting a wide variety of traits in poplar, but it can also be used to map the co-functional linkages to other woody plants with high efficiency. PoplarGene can also accept guide genes from Arabidopsis, the most extensively studied plant species, which will greatly facilitate investigations of the less-studied plant poplar. PoplarGene will continue to be improved. When more published data are available for poplar research, literature-based network inference methods will be incorporated into PoplarGene. In summary, we believe that PoplarGene will serve as a highly useful tool for the scientific community, facilitating studies of poplar and other woody plants.

## Methods

### Gold standard gene pairs for machine learning

To construct and evaluate the network, gold standard co-functional gene pairs were generated from four sources of annotated sets of *P*. *trichocarpa*: Biological Process of Gene Ontology (GO-BP)^23^, Kyoto Encyclopedia of Genes and Genomes (KEGG) pathways^24^, MapMan metabolic pathways^25^ and PoplarCyc metabolic pathways^26^. The positive gene pairs were derived by pairing genes sharing at least one functional annotation in each annotation set, while the negative pairs were obtained by pairing genes that do not share any functional annotation terms. In the GO annotation set, gene pairs sharing annotation from the same GO term were considered to be functionally linked, while the pairs of annotated genes not sharing any GO terms were treated as negative pairs^49^. For example, the gene Potri.015G088100 and Potri.011G023800 represent a positive pair, sharing GO terms “GO:0006281: DNA repair”, “GO:0006310: DNA recombination”. The gene Potri.004G061800 and Potri.010G136500 is a positive pairs, sharing GO term “GO:0016567: protein ubiquitination”. The gene Potri.003G183000 (annotated with GO:0005216, GO:0016020, GO:0006811 and GO:0055085) and Potri.004G061800 (annotated with GO:0016567, GO:0004842, GO:0000151 and GO:0005515) do not share any term and represent a negative example. Among the GO-BP terms, since terms above level 2 are too general and terms below level 11 are too specific, we used the terms belonging to levels 2 through 10 to optimize annotation specificity and comprehensiveness^37^. If a term/pathway has too many annotated genes, there will be too many gene pairs generated from a single term/pathway, which may cause functional bias towards the term/pathway^12^,^50^. For instance, among the Poplar BFGR GO-BP terms, six top broad GO-BP terms will generate 1,984,503 positive linkage pairs, which account for ~92% of total 2,155,797 positive linkage pairs (based on all 341 Poplar BFGR GO-BP terms), thereby leading to strong bias toward these broad terms. It is the same case for KEGG pathway, Mapman pathway and PoplarCyc pathway. Thus, to reduce the training bias, the terms/pathways containing too many genes were ignored in the gold standard gene pair construction. The ignored terms/pathways, which typically contains >300 genes, are listed in Supplementary Table S1. As a result, GO-BP generated 171,294 positive and 7,300,003 negative gene pairs, covering 3,877 (~9.4%) *P*. *trichocarpa* genes. For KEGG pathway (Release 76.0) analysis, after ignoring the largest terms and broad-concept terms, 440,925 positive and 12,991,275 negative pairs were obtained, covering 5,198 (12.6%) poplar genes. The gold standard gene pairs from MapMan metabolic pathways included 318,481 and 51,307,487 positive and negative gene pair (10,162, ~24.6% of *P*. *trichocarpa* genes), respectively. For PoplarCyc (version 3.0), since the largest pathways contain the fewest annotated genes, no terms were ignored, and 118,243 positive and 10,844,660 negative gene pairs were obtained for 4,683 genes (11.3% of *P*. *trichocarpa* genes). Finally, after merging the four types of gold standard gene pairs, a total of 961,462 positive and 72,756,688 negative gold standard gene pairs were obtained, covering 15,677 (~38%) *P*. *trichocarpa* genes.

### Function links inferring framework and data integration

The functional linkages derived from different data sets have different levels of confidence due to variations in the internal measurements of different types of data sets. To unify the dataset-intrinsic scores and to integrate heterogeneous data into a composite network, a common Bayesian scoring framework, LLS^37^, was initially used to measure the functional linkages between two genes in each dataset, which was defined as:


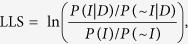


where P(I|D) and P(~I|D) represent the frequencies of gold standard positive and negative gene pairs observed in the corresponding dataset (D), and P(I) and P(~I) are the frequencies of all positive and negative gold standard gene pairs, respectively. To avoid over-fitting bias, 0.632 bootstrapping, which provides a robust estimate of classifier accuracy and is appropriate for poorly annotated genomes^51^, was used to calculate LLS values^37^.

For each dataset, the gene pairs were ranked by their respective continuous intrinsic scores (mutual information, correlation coefficient, gene distance and so on), and LLS for bins with equal numbers of ranked gene pairs were calculated. Regression models were then constructed based on these LLS values, and the set of mean continuous scores for bins was used to map the intrinsic score of each gene pair to LLS values in a continuous manner^28^.

### Linkages data integration framework

The functional links in each dataset were generated; a functional link could be observed in multiple datasets with different LLS values. Because the datasets were not fully independent, the weighted sum (WS)^28^, which is a modification of the native Bayesian, was used to integrate the linkages derived from various dataset. WS is defined as:


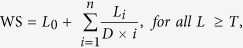


where L is the LLS value (*L*_0_ is the largest LLS among the datasets supporting the link), and i (in L_i_) is the rank index number of the remaining LLS values of the link. D is the weight factor, which ranges from 1 to + ∞, and T is the minimum threshold of LLS. LLS values above the threshold were considered in order to exclude noisy, low-scoring linkages. Systematic testing was conducted to select the optimal values of D and T in order to maximize overall performance, which was measured as the area under a plot of LLS versus the number of gene pairs in the network^37^.

### Functional links inferred from genomic contexts

The two most widely used genomic context methods, Phylogenetic Profiling^52^,^53^,^54^ and Gene Neighborhood^55^,^56^,^57^, which have shown reasonable performance for interring functional linkages in Arabidopsis and rice, were applied to infer functional associations in poplar. Phylogenetic Profiling is a method that uses similarity of evolutionary co-occurrence patterns among large numbers of species to infer functional couples. First, BLASTP was used to align all *P*. *trichocarpa* protein sequences against the unique representative complete genomes in each of the three domains of life (1,188 Bacteria species, 159 Archaea species and 434 Eukaryota species), respectively. The species with the largest genomes were chosen as the unique representative species in each genus. Second, the best BLAST hit was used to construct a phylogenetic profile matrix for each domain of life, and the similarity between two profiles was then measured by mutual information (MI)^15^. The functional linkages generated in the three domains of life were integrated into a single network by the weighted-sum framework mentioned above. Meanwhile, two complementary Gene Neighborhood algorithms, physical distance based neighborhood^56^ and probability-based neighborhood^55^, were used to infer functional links separately, which were integrated into a single network by the weighted-sum framework as well.

### Functional links inferred from the co-occurrence protein domains

The protein domain is the functional subunit of a protein. Proteins sharing a similar set of domains may perform similar functions^49^. Rare domains are more closely related to specific functions than common domains^49^. Using the protein PFAM domain annotation^58^, domain occurrence profiles (3,375 unique domains) were generated for all protein sequences, with the inverse of the domain frequency in the *P*. *trichocarpa* proteome indicating the presence of the corresponding domain and 0 indicating its absence. This type of weighted scoring gives more weight to rare domains. The mutual information was then calculated to determine the significance of domain co-occurrence within the profile matrix to infer functional linkages.

### Inferring functional linkages from associalogs

Associalogs are defined as conserved functional linkages that are transferred from other organisms by orthology^37^. The functional linkages were transferred to *P*. *trichocarpa* genes from AraNet v2 (*Arabidopsis thaliana*)^12^, WormNet v3 (*Caenorhabditis elegans*)^18^, HumanNet v1 (*Homo sapiens*)^59^, FlyNet v1 (*Drosophila melanogaster*)^13^, RiceNet v2 (*Oryza sativa*)^32^ and YeastNet v3 (*Saccharomyces cerevisiae*)^60^. All transferred functional linkages were scored by InParanoid weighted LLS (IWLLS)^16^, which is defined as:


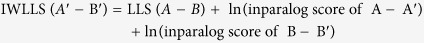


where A and B are poplar genes and A′ and B′ are orthologous genes from other organisms. An InParanoid score is calculated by multiplying two inparalog scores, i.e., those of the poplar gene and the orthologous gene in another organism (A − A′/B − B′), which are generated from the InParanoid algorithm^21^.

### Inferring functional linkages based on co-expression patterns

Functionally associated genes tend to be co-expressed under various conditions^35^. High dimensional microarray data have been broadly used to infer co-functional links based on correlations in gene co-expression patterns. First, 32 microarray datasets with no less than 12 samples were obtained from Gene Express Omnibus (GEO) in May 2015. Datasets with fewer than 12 samples were excluded because co-functional links inferred by correlation with small sample sizes may be promiscuous. Second, expression profile vectors for each gene across microarray samples were generated for each GEO dataset. Finally, Pearson Correlation Coefficient (PCC) values were calculated between each pair of expression profile vectors to measure the co-expression correlation. Only gene pairs with PCC values that were statistically significant at the 99% confidence level (t-test) were retained. After filtering the dataset with lower co-expression correlation, 22 co-expression networks were obtained, which were further integrated into a single co-functional network via the weighted-sum framework.

## Additional Information

**How to cite this article**: Liu, Q. *et al.* PoplarGene: poplar gene network and resource for mining functional information for genes from woody plants. *Sci. Rep.* **6**, 31356; doi: 10.1038/srep31356 (2016).

## Supplementary Material

Supplementary Information

## Figures and Tables

**Table 1:** Summary of the PoplarGene network and 23 network components.

(Network source) description	#Nodes (coding genes coverage, %)	#Links
PoplarGene network	29 049 (70.3)	1 967 631
(PT-CX) Co-expression network of Poplar genes using microarray experiments	7 930 (19.2)	282 144
(PT-DC) Protein domains co-occurrence between two Poplar genes	3 022 (7.3)	27 096
(PT-GN) Neighborhood conservation of Poplar genes in prokaryotic genomes	8 881(21.5)	213 509
(PT-PG) The similarity of phylogenetic profile between Poplar genes	11 623 (28.1)	305 305
(AT-CC) Transfer of co-citation links in A. thaliana orthology network	7 243 (17.5)	65 474
(AT-CX) Transfer of co-expression links in A. thaliana orthology network	18 290 (44.2)	418 367
(AT-HT) Transfer of high-throughput PPI in A. thaliana orthology network	3 442 (8.3)	6 390
(AT-LC) Transfer of literature curated PPI in A. thaliana orthology network	2 290 (5.5)	3 952
(CE-CX) Transfer of co-expression links in C. elegans orthology network	6 273 (15.2)	104 876
(CE-HT) Transfer of high-throughput PPI in C. elegans orthology network	1 781 (4.3)	5 296
(CE-LC) Transfer of co-citation links in C. elegans orthology network	1 243 (3.0)	4 873
(DM-CX) Transfer of co-expression links in D. melanogaster orthology network	1 719 (4.2)	15 033
(DM-HT) Transfer of high-throughput PPI in D. melanogaster orthology network	1 272 (3.1)	3 120
(DM-LC) Transfer of literature curated PPI in D. melanogaster orthology network	104 (0.3)	183
(HS-CX) Transfer of co-expression links in H. sapiens orthology network	5 100 (12.3)	88 318
(HS-HT) Transfer of high-throughput PPI in H. sapiens orthology network	1 661(4.0)	5 716
(HS-LC) Transfer of literature curated PPI in H. sapiens orthology network	5 176 (12.5)	55 102
(OS-CX) Transfer of co-expression links in O. sativa orthology network	3 187 (7.7)	30 275
(OS-LC) Transfer of literature curated PPI in O. sativa orthology network	28 (0.1)	80
(SC-CC) Transfer of co-citation links in S. cerevisiae orthology network	6 396 (15.5)	146 710
(SC-CX) Transfer of co-expression links in S. cerevisiae orthology network	4 866 (11.8)	141 350
(SC-HT) Transfer of high-throughput PPI in S. cerevisiae orthology network	5 486 (13.3)	274 397
(SC-LC) Transfer of literature curated PPI in S. cerevisiae orthology network	6 086 (14.7)	147 250
