# Supplementary material for: PoplarGene: poplar gene network and resource for mining functional information for genes from woody plants
Source: Sci Rep. 2016 Aug 12;6:31356. doi: 10.1038/srep31356 (PMC4981870; doi:10.1038/srep31356)
Supplement: Supplementary Information [file srep31356-s1.pdf]

Supplementary Information

PoplarGene: poplar gene network and resource for mining functional information for genes from woody plants

Qi Liu, Changjun Ding, Yanguang Chu, Jiafei Chen, Weixi Zhang, Bingyu Zhang, Qinjun Huang, Xiaohua Su

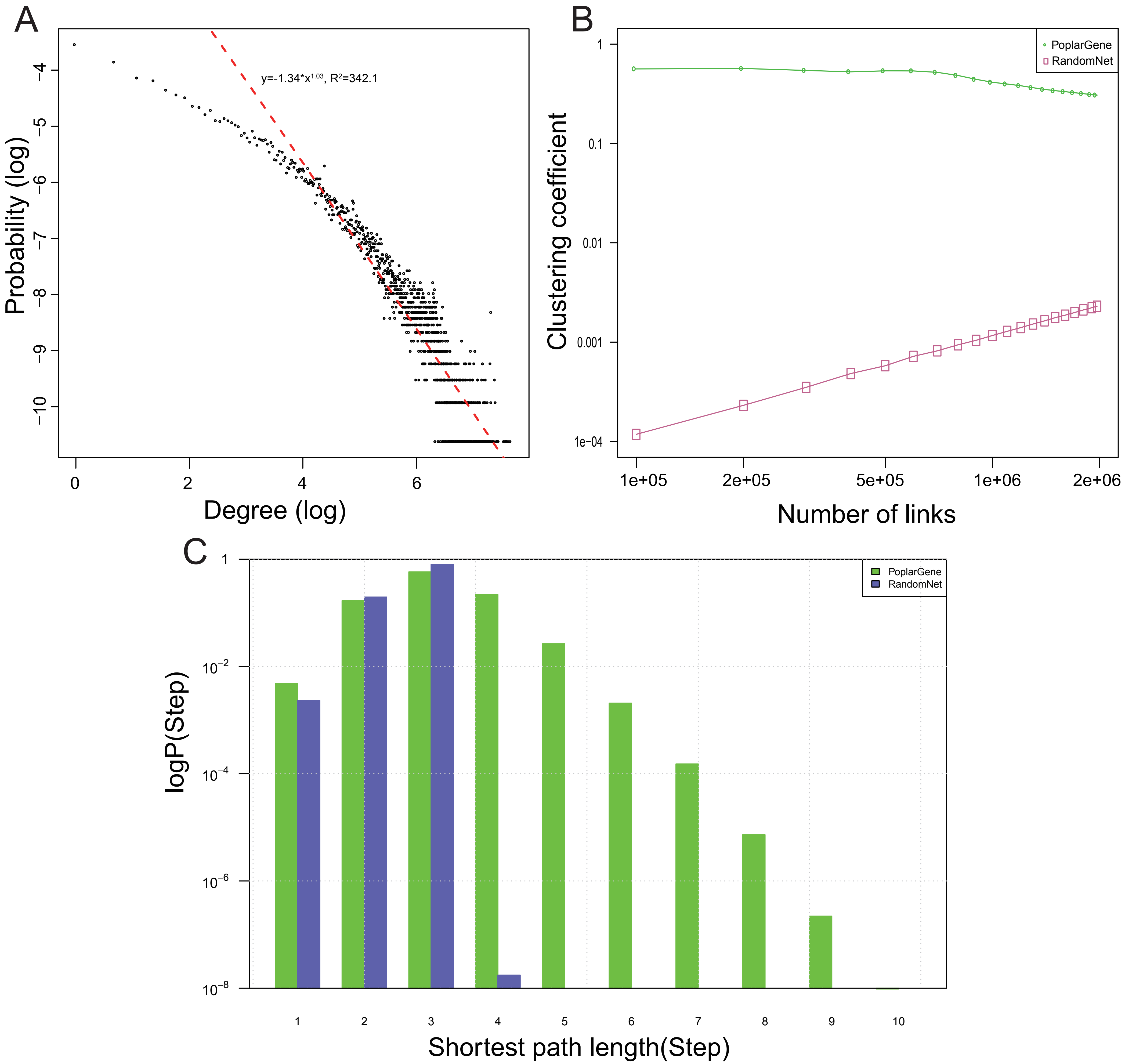

Figure S1. Network features assessment for the PoplarGene network. (A) The PoplarGene network exhibits a scale-free property and a high degree of modularity, indicating that the network captures diverse biological processes and pathways. (B) PoplarGene exhibits a significantly higher network-clustering coefficient than a randomized network. (C) PoplarGene exhibits a significantly non-random distribution of shortest path lengths between two genes, indicating that many regional structures (functional gene modules) of the PoplarGene network are separated from one another.

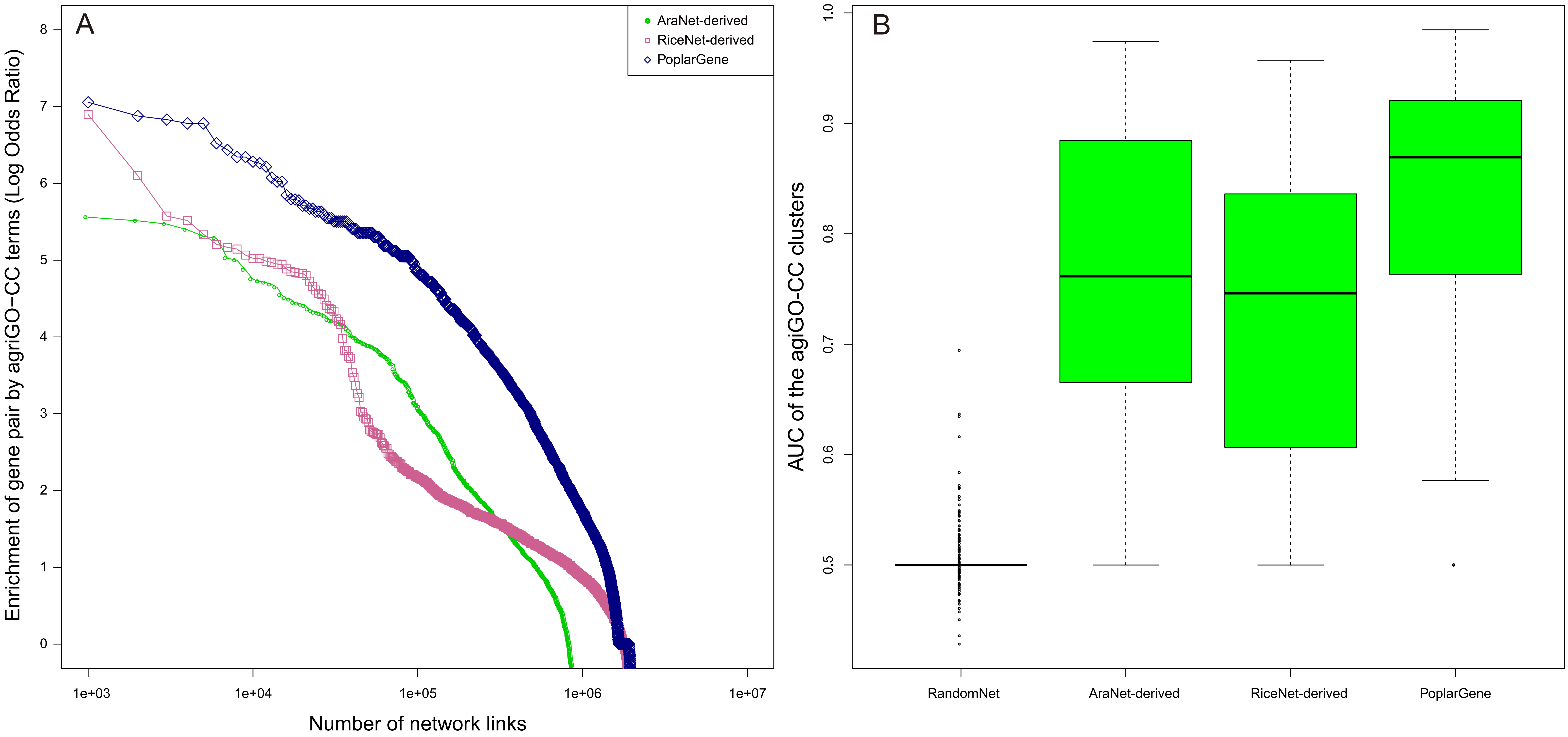

Figure S2. Precision-recall analysis comparing the PoplarGene network to the AraNet-derived network and the RiceNet-derived network (A) and network predictive power analysis (B) based on the agriGO-CC-derived benchmark set.

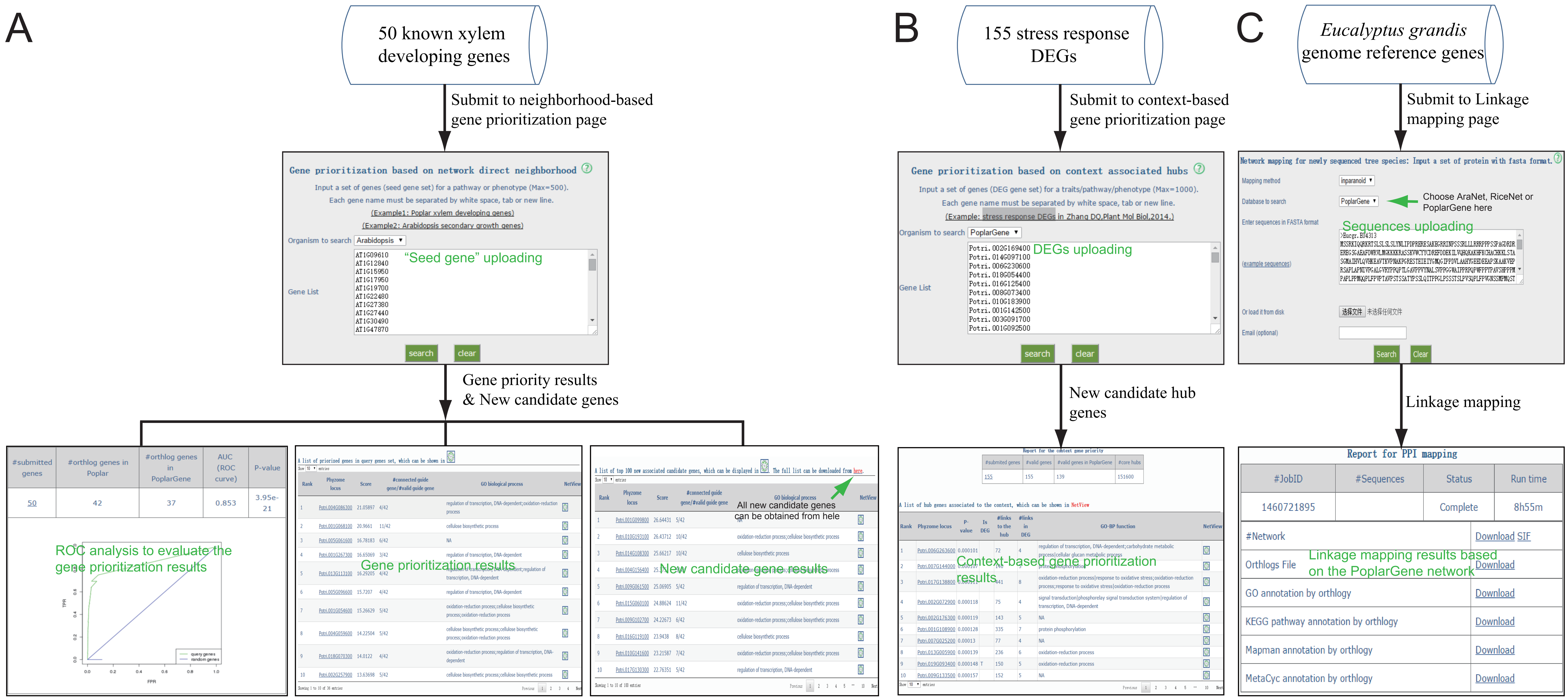

Figure S3. Workflows for the case studies. (A) Neighborhood-based gene prioritization. (B) Context-based gene prioritization. (C) Network mapping to *Eucalyptus grandis*.

Table S1. Annotation terms/pathways ignored during the gold-standard gene pairs construction.

**Annotation set**	**Ignored terms**
GO-BP	GO:0006468: protein phosphorylation
	GO:0055114: oxidation-reduction process
	GO:0006355: regulation of transcription, DNA-dependent
	GO:0055085: transmembrane transport
	GO:0005975: carbohydrate metabolic process
	GO:0006412: translation
KEGG pathway	pop03010: Ribosome
	pop04075: Plant hormone signal transduction
	pop01200: Carbon metabolism
	pop01212: Fatty acid metabolism
	pop01210: 2-Oxocarboxylic acid metabolism
	pop01230: Biosynthesis of amino acids
	pop01220: Degradation of aromatic compounds
Mapman pathway	29.5.11.4: protein degradation ubiquitin E3 RING
	27.3.99: RNA regulation of transcription unclassified
	29.4.1.57: protein postranslational modification kinase receptor like cytoplasmatic kinase VII
	30.2.17: signaling receptor kinases DUF 26
	30.2.11: signaling receptor kinases leucine rich repeat XI
	27.3.67: RNA regulation of transcription putative transcription regulator
	27.3.25: RNA regulation of transcription MYB domain transcription factor family
	20.2.1: stress abiotic heat
	29.5.11.4.3.2: protein degradation ubiquitin E3 SCF FBOX
	20.1.7: stress biotic PR-proteins
	27.3.6: RNA regulation of transcription bHLH, Basic Helix-Loop-Helix family
	29.3.4.99 protein targeting secretory pathway unspecified
PoplarCyc pathway	NA
